# A Study of The Local Toxicity of Agents Used for Variceal Injection Sclerotherapy

**DOI:** 10.1155/1989/79120

**Published:** 1989

**Authors:** C. S. Robertson, C. Womack, K. Robson, D. L. Morris

**Affiliations:** 1 Department of Surgery University Hospital Nottingham NG7 2UH UK; 2 Department of Pathology University Hospital Nottingham NG7 2UH UK

## Abstract

Injection sclerotherapy is widely used in the treatment of oesophageal varices. However, few studies have compared the local toxicity of sclerosant agents which may be important if serious local complications are to be avoided.

In this study the depth of injury caused by submucosal injection of increasing concentrations of sodium tetradecyl sulphate, polidocanol, 5% ethanolamine oleate and 5% varicosid in rabbits stomach, has been compared by histopathological examination.

Macroscopic ulceration was seen in 14.6% of injection sites. Increasing concentrations of sodium tetradecyl sulphate and polidocanol produced increasingly extensive microscopic inflammation. Five percent varicosid caused more inflammation than 5% ethanolamine and only 3% polidocanol and 5% varicosid caused full thickness inflammation. Only 5% ethanolamine produced inflammation consistently confined to the mucosa and submucosa.

On the basis of this study we feel that 5% ethanolamine is the most suitable agent for injection sclerotherapy.


*HPB Surgery* 1989, Vol. 1, pp. 149–154
Reprints available directly from the publisher
Photocopying permitted by license only
© 1989 Harwood Academic Publishers GmbH
Printed in Great Britain

### A STUDY OF THE LOCAL TOXICITY OF AGENTS USED FOR VARICEAL INJECTION SCLEROTHERAPY

C.S. ROBERTSON**, C. WOMACK*, K. ROBSON* and D.L. MORRIS*

*Departments of Surgery & *Pathology, University Hospital, Nottingham NG7 2UH, UK*

*(Received 18 July 1988)*

Injection sclerotherapy is widely used in the treatment of oesophageal varices. However, few studies have compared the local toxicity of sclerosant agents which may be important if serious local complications are to be avoided.

In this study the depth of injury caused by submucosal injection of increasing concentrations of sodium tetradecyl sulphate, polidocanol, 5% ethanolamine oleate and 5% varicosid in rabbits stomach, has been compared by histopathological examination.

Macroscopic ulceration was seen in 14.6% of injection sites. Increasing concentrations of sodium tetradecyl sulphate and polidocanol produced increasingly extensive microscopic inflammation. Five percent varicosid caused more inflammation than 5% ethanolamine and only 3% polidocanol and 5% varicosid caused full thickness inflammation. Only 5% ethanolamine produced inflammation consistently confined to the mucosa and submucosa.

On the basis of this study we feel that 5% ethanolamine is the most suitable agent for injection sclerotherapy.

KEY WORDS: Injection sclerotherapy, oesophageal varices, sclerosants.

### INTRODUCTION

Endoscopic injection sclerotherapy has a well established place in the management of acute variceal haemorrhage and in the more long-term treatment of oesophageal varices[1,2,3]. However, the local effects of sclerosant agents are the least well studied area of variceal sclerotherapy. Whilst there is some evidence that intravascular injection is more effective than the paravariceal technique[4], some submucosal extravasation can be expected even with intravariceal injection and this probably accounts for the significant incidence of local ulceration seen[5]. Few studies have compared the local inflammatory response to sclerosant agents and there is little scientific basis on which to choose the best sclerosant. The local toxicity of several commonly used sclerosants has been compared in this study in which the depth and severity of inflammation caused by sclerosant injection in rabbits stomach has been assessed by histopathological examination.

### METHOD

Twenty rabbits underwent laparotomy and gastrotomy under general anaesthesia (Hypnorm [Janssen Ltd] 0.25 ml/kg I.M. + $N_20/0_2$/Halothane). Each rabbit

**Address correspondence to: Mr C. S. Robertson, Department of Surgery, University Hospital, Nottingham, NG7 2UH

received 3 submucosal injections of sclerosant at different sites within upper stomach. The injection sites were marked by indentifiable, non-absorbable sutures.

Four groups were studied: Group A (n=10) received 0.1 ml of 1%, 2% and 3% sodium tetradecyl sulphate (STD) at one of the 3 injection sites; Group B (n=5) received 0.1 ml of 5% ethanolamine oleate (EO) at each of the 3 injections sites. Group C (n=5) received 0.1 ml of 0.5%, 2% and 3% polidocanol at 1 of 3 injection sites. Group D (n=5) received 0.1 ml of 5% varicosid (sodium morrhuate) at each of the 3 injection sites. 0.1 ml was the chosen volume of sclerosant per injection site in the rabbit because it is approximately the ratio of sclerosant to body weight used in patients (eg 0.05 ml/kg body weight). Injections were judged to be into the submucosa when an easily identifiable submucosal bleb was raised.

The gastrotomy was closed and the rabbits recovered. One week following injection the rabbits were sacrificed and the stomach removed for histopathological examination. Representative blocks were cut from each of the marked injection sites and in addition control blocks were taken from areas of the stomach inbetween injection sites.

Standard haematoxylin and eosin stained sections were prepared and examined by one histopathologist, who was unaware of the sclerosant used. To assess the degree of local tissue reaction sections were scored according to the depth of inflammation present on a scale of 1–4 (Table 1).

*Statistical Analysis* was by Fisher's Exact Test and a value of $p < 0.05$ was taken to denote statistical significance.

**Table 1** Pathologists score of the depth of inflammation at the injection site

| *Pathologists score* | *Depth of inflammation at the injection site* |
|---|---|
| 1 = | Mucosa only |
| 2 = | Mucosa + submucosa |
| 3 = | 1 +2+muscularis propria |
| 4 = | Full thickness of stomach wall/perforation |

### RESULTS

Whilst all injection sites appeared inflammed to the naked eye, macroscopic ulceration was only seen in 11 of the 75 injection sites (14.6%).

The results of histopathological examination are summarised in Table 2. Collapsing the data, by combining groups 1 with 2 and 3 with 4, allowed statistical analysis using Fisher's Exact Test. The depth of injury increased with increasing concentrations of S.T.D. (1% v 2% $p < 0.01$, 1% v 3% $p < 0.01$, 2% v 3% $p < 0.01$) and polidocanol (0.5% v 2% p = NS; 2% v 3% p = NS; 0.5% v 3% $p < 0.01$) but only one of the 3% polidocanol injection sites showed full thickness inflammation. 5% varicosid caused more inflammation than 5% ethanolamine oleate and none of the E.O. injection sites showed inflammation of the muscularis propria in contrast one of the 5% varicosid injection sites showed full thickness inflammation.

Control blocks showed only an organising peritoneal reaction consistent with a gastrotomy.

**Table 2** Summary of histopathological findings.

| Groups | | Macroscopic ulceration | Depth of inflammation at injection sites | | | |
|---|---|---|---|---|---|---|
| | | | 1 | 2 | 3 | 4 |
| A STD (n = 10) | 1% | 0 | 5 | 5 | 0 | 0 |
| | 2% | 0 | 1 | 4 | 5 | 0 |
| | 3% | 4 | 0 | 0 | 10 | 0 |
| B (n = 5) | 5% Ethanolamine oleate | 0 | 0 | 15 | 0 | 0 |
| C (n = 5) | Polidocanol | | | | | |
| | 0.5% | 0 | 1 | 4 | 0 | 0 |
| | 2% | 1 | 0 | 3 | 2 | 0 |
| | 3% | 3 | 0 | 0 | 4 | 1 |
| D (n = 5) | 5% Varicosid | 3 | 0 | 4 | 10 | 1 |

| | | | |
|---|---|---|---|
| 5% Ethanolamine (Fisher's Exact Test) | v 1% | STD | $p = NS$ |
| | v 2% | STD | $p < 0.01$ |
| | v 3% | STD | $p < 0.01$ |
| | v 0.5% | Polidocanol | $p = NS$ |
| | v 2% | Polidocanol | $p < 0.05$ |
| | v 3% | Polidocanol | $p < 0.01$ |
| | v 5% | Variscosid | $p < 0.01$ |

### DISCUSSION

The choice of sclerosant used for variceal injection sclerotherapy varies in different countries and different units but in the UK STD and EO are the 2 most frequently used, whereas in other parts of Europe hydroxypolyethoxy dodecanoic acid (Polidocanol) and varicosid (sodium morrhuate) are commonly used.

A rational choice of sclerosant would take into account its efficacy, local toxicity, ease of injection and systemic effects eg anaphylaxis and fever. Ideally a sclerosant would cause thrombosis of the varix with few local complications. Ulceration, stricture formation and, rarely, perforation are local complications thought to be the direct result of the intensity of local inflammation. Some authors[6] recommend paravasal, submucosal injections of sclerosant to achieve oesophageal wall sclerosis, thus burying the varices in a thick protective fibrous coat. This technique may lead to more local complications and in addition during intended intravariceal injection some extravasation is likely to occur[7]. We therefore felt it was worthwhile to try and identify the degree of local inflammation caused by different sclerosants.

Whilst we are aware that gastric mucosa is not identical to oesophageal mucosa, we were specifically interested in the local tissue damage caused by the sclerosants. Ulceration may occur over a variable time period but we felt that serial analysis would have required the sacrifice of large numbers of rabbits.

Macroscopic ulceration was only seen in 14.6% of our injection sites however it has been claimed that oesophageal ulceration is a necessary accompaniment rather than an unavoidable complication of sclerotherapy[5]. Both 3% polidocanol and 5% varicosid caused inflammation of the muscle layer and full thickness inflammation.

This is probably undesirable as it may contribute to the small but very real risk of oesophageal stricture formation following injection sclerotherapy[8]. In a study of the effect of sclerosants using rat femoral vein[9], Blenkinsop has shown that 1 and 3% STD were superior to EO whilst STD and EO were equally ulcerogenic when injected subcutaneously. Silpa *et al.* found in a study of efficacy and safety of sclerosing agents that STD was effective but there was a 60% incidence of oesophageal ulceration[10].

Gibbert *et al.* in another comparative study of sclerosants found 3% S.T.D. to be associated with less oesophageal ulceration than sodium morrhuate[11]. STD is less viscous than EO and therefore more easily injected and it is also less irritant to the eye if spillage occurs while injecting under high pressure[12]. In addition, STD may have a more marked effect on increasing blood viscosity than EO[13]. However, only 5% E.O. injection sites consistently produced inflammation confined to the mucosa and submucosa and we therefore feel that it is the most suitable agent for injection sclerotherapy of oesophageal varices.

### *Acknowledgement*

We wish to thank the staff of the animal unit and the pathology department for their help.

### *References*

1. Terblanche, J., Northover, J.M.A., Bornman, P. et al (1979). A prospective evaluation of injection sclerotherapy in the treatment of acute bleeding from oesphageal varices. *Surgery;* **85:** 239–245.
2. Terblanche, J., Northover, J.M.A., Bornman P. *et al* (1979). A prospective controlled trial of sclerotherapy in the long term management of patients after oesophageal variceal bleeding. *Surg Gyn & Obs;* **148:** 323–333.
3. MacDougall, B.R.D., Westaby, D., Theodossi, A., Dawson, J.L., Williams, R. (1982). Increased long term survival in variceal haemorrhage using injection sclerotherapy. *Lancet;* **1:** 124–127.
4. Rose, J.D.R., Crane, M.D., Smith, P.M. (1983). Factors affecting successful endoscopic sclerotherapy for oesophageal varices. *Gut;* **24:** 946–949.
5. Sarin, S.K., Nanda, R, Vij, J.C., Anand, B.S. (1986). Oesophageal ulceration after sclerotherapy – a complication or an accompaniment? *Endoscopy;* **18:** 44–45.
6. Paquet, K.J. (1983). Endoscopic paravariceal injection sclerotherapy of the oesophagus. Indications, technique, complications: results of a period of 14 years. Gastrointest Endosc; 29: 310–315.
7. Rose, J.D.R., Crane, M.D., Smith, P.M. (1982). Studies in the treatment of bleeding oesophageal varices by fibre endoscopic sclerotherapy. *Gut;* **23:** 439.
8. Sorenson, T.I.A., Burcharth, F., Pedersen, M.L., Findahl, F. (1984). Oesophageal stricture and dysphagia after endoscopic sclerotherapy for bleeding varices. Gut; 25: 473–477.
9. Blenkinsop, W.K. (1968). Comparison of tetradecyl sulphate of sodium with other sclerosants in rats. *Br J Exp Path;* **49:** 197–207.
10. Silpa, M.L., Jensen, D.M., Machicado, G.A., Tapia, J.I., Beilin, D.B. (1982). Efficacy and safety of agents for variceal sclerotherapy. *G.I. Endos;* **28:** 152–153 (A).
11. Gibbert, V., Feinstat, T., Burns, M., Trudeau, W. (1982). A comparison of the sclerosing agents sodium tetradecyl sulfate and sodium morrhuate in endoscopic injection sclerosis of esophageal varices. *G.I. Endos;* **28:** 147 (A).
12. Smith, P.M. (1986). Therapeutic upper gastrointestinal endoscopy. *Br. Med Bull;* **42:** 249–256.
13. Rose, J.D.R. (1983). Practical problems of oesophageal sclerotherapy. The Hopkins Endoscopy Prize essay.

*Accepted by S. Bengmark on 21 September 1988*

### INVITED COMMENTARY

Endoscopic sclerotherapy is now established as the "best buy" in the management of the acute bleeding episode from oesophageal varices. A programme of chronic injection sclerotherapy is also effective in reducing the risk of recurrent haemorrhage. However many points of sclerotherapy technique remain controversial: the use of anaesthesia or sedation, flexible or rigid instrument, intra- or para-variceal injection, compression or not, site of injection, ideal treatment intervals and the best sclerosant. This paper by Robertson and colleagues deals with this last point.

There is a wide variety of sclerosants in current use and this aspect of management has received remarkably little investigation. In the case of intravariceal injections the ideal sclerosant would be one which causes variceal thrombosis with minimal inflammation of the surrounding tissue. However realistically even those who attempt intravascular injection acknowledge that sclerosant goes outside the vein in about 20 per cent of instances in the case of large varices and in a much higher percentage of patients with small varices[1]. Where paravariceal injection is preferred the ideal sclerosant should cause sufficient inflammation to cause thickening and fibrosis of the mucosa with a view to protecting the underlying varices. Some centres consider superficial ulceration of the mucosa of the lower oesophagus to be desirable and an end point to sclerotherapy[2,3]. I am unhappy with this viewpoint since ulceration carries not only the risk of further bleeding but also subsequent oesophageal stricture secondary to fibrosis. More and more centres are now reporting a significant incidence of oesophageal strictures after courses of sclerotherapy.

There are a number of sclerosants in common use. Sodium tetradecyl sulphate (STD) is widely used in the USA. Polidocanol is the favoured agent in Europe. Absolute alcohol is used in India and is claimed to be both economical and effective. Ethanolamine oleate (EO) is at present the most commonly used sclerosant in the British Isles, Japan and South Africa. This paper by Robertson and colleagues is useful in assessing the relative merits of the most commonly used agents. Although the study is experimental and used submucosal injections in the rabbits' stomachs, the results are probably relevant to the clinical situation. Their experimental work however cannot allow the authors to conclude that EO "is the most suitable agent for injection sclerotherapy of oesophageal varices." They do show that it gives less serious inflammation and is therefore less likely to produce perforation, ulceration or stricture but this experimental study cannot demonstrate that the agent is effective in reducing bleeding from oesophageal varices. In another animal experiment Jansen and colleagues found EO and STD to be similarly effective in producing thrombosis but the ulceration rate with STD was much higher, namely, 40 per cent[4]. In a recent controlled trial involving 45 patients with cirrhosis Kitano and colleagues showed that EO was both safer and more effective and 2 per cent STD for intravariceal injection[3]. There was significantly less post-injection bleeding with EO and the incidence of oesophageal ulceration was only nine per cent compared to 43 per cent with STD. They used the intravariceal route of injection and a number of trials suggest it is the more effective route[1,5]. Nevertheless the results obtained by Paquet using the paravariceal route are among the best in the world[6]. We have been using intravariceal EO for injection sclerotherapy in Belfast for thirty years[7]. More than 400 patients have had in excess of 800 episodes of injection sclerotherapy. We do not have a true figure for the incidence of oesophageal ulceration since we tend to do the injections at monthly intervals and therefore rarely see ulceration. However we can say that in the whole period no patient with cirrhosis developed a symptomatic oesophageal stricture. Two patients with extrahepatic block, each receiving more than 12 episodes of sclerotherapy, developed strictures – both were relieved with only one dilatation.

From the evidence currently available we feel that the intravariceal injection of EO is probably the "best buy" where injection sclerotherapy is used for the control of bleeding oesophageal varices.

### *References*

1. Rose, J.D.R., Crane, M.D., Smith, P.M. (1983). Factors affecting successful endoscopic sclerotherapy for oesophageal varices. *Gut* **24:** 946–949.
2. Sarin, S.K., Nanda, R., Vij, J.C., Anand, B.S. (1986). Oesophageal ulceration after sclerotherapy – a complication or an accompaniment. *Endoscopy* **18:** 44–45.
3. Kitano, S., Iso, Y., Yamaga, H., Hashizume, M., Higashi, H., Sugimachi, K. (1988). Trial of sclerosing agents in patients with oesophageal varices. *ritish J Surgery* **75:** 751–753.
4. Jensen, D.M., Machicado, G.A., Silpa, M. (1986). Esophageal varix haemorrhage and sclerotherapy – animal studies. *Endoscopy* **18:** 18–22.
5. Sarin, S.K., Nanda, R., Sachdev, G., Chari, S., Anand, B.S., Broor, S.L. (1987). Intravariceal versus paravariceal sclerotherapy. A prospective controlled trial. *Gut* **28:** 657–662.
6. Paquet, K.J. (1983). Endoscopic paravariceal injection sclerotherapy of the esophagus – indications, technique, complications. Results of a period of fourteen years. *Gastrointestinal Endoscopy* **29:** 310–315.
7. Johnston, G.W., Rodgers, H.W. (1973). A review of 15 years' experience in the use of scleortherapy in the control of acute haemorrhage from oesophageal varices. *British J Surgery* **60:** 797–800.

George W. Johnson
Royal Victoria Hospital
Belfast BT12 6BA, UK

